# TOPSIS Multi-Attribute Decision-Making Model Utilizing Novel Distance Measure of Picture Fuzzy Sets and Its Application in Power Battery Recycling Evaluation

**DOI:** 10.3390/e28060620

**Published:** 2026-05-31

**Authors:** Supan Yang, Haiping Ren, Xiaoqing Huang

**Affiliations:** 1 School of Business, Jiangxi University of Science and Technology, Nanchang 330013, China; 6120241570@mail.jxust.edu.cn (S.Y.); 9520060004@jxust.edu.cn (H.R.); 2 Department of Basic Subjects, Jiangxi University of Science and Technology, Nanchang 330013, China

**Keywords:** picture fuzzy set, multi-attribute decision-making, distance measure, TOPSIS, power battery

## Abstract

The recycling of power batteries is a key measure for improving the new energy industry chain and achieving green circular economy goals. However, the process of evaluating and selecting recycling schemes is influenced by multiple complex factors and often involves a significant amount of ambiguous and uncertain decision-making information. As an important extension of intuitionistic fuzzy sets, picture fuzzy sets characterize fuzzy information through three distinct dimensions: membership, neutrality, and non-membership. This three-dimensional structure offers unique advantages in addressing uncertain and ambiguous decision-making problems, where traditional fuzzy sets may lose valuable information. Drawing on the Bray–Curtis distance measure, this paper proposes a novel picture fuzzy distance measure that captures differences across all three dimensions more comprehensively. By combining the weighted form of the proposed picture fuzzy distance measure with the classical TOPSIS method, a new multi-attribute decision-making model is established under the picture fuzzy framework. The effectiveness and feasibility of the proposed method are demonstrated through a case study on the recycling of power batteries for electric vehicles. A sensitivity analysis of relevant parameters is conducted, confirming the stability of the model against variations in parameter settings. Comparative results indicate that the proposed novel picture fuzzy distance measure exhibits superior robustness compared to existing similar distance measures. Furthermore, the constructed decision-making model can provide reliable and practical support for uncertain multi-attribute decision-making problems in real-world applications.

## 1. Introduction

With the rapid and high-quality development of electric vehicle (EVs) in recent years, the number of such vehicles in China has exceeded 40 million. As the core component of EVs, power batteries (PBs) typically have a service life of 5–8 years. It is estimated that the volume of the end-of-life PBs in 2026 will exceed 1.5 million tons. The recycling of PBs has become a crucial step in ensuring the sustainable development of the industry and putting the principles of a circular economy into practice, while also placing higher demands on recycling companies in terms of environmental compliance, technical standards and carbon reduction benefits. Currently, the academic community has conducted extensive research on the PB recycling supply chain, focusing on multiple dimensions such as interactions among supply chain actors, policy interventions, optimization of recycling models, and decision-making (DM) methods, resulting in a wealth of research findings. Existing research primarily explores the various stakeholders, policy scenarios, and core DM issues within the PB recycling supply chain, providing a solid theoretical foundation and practical reference for future studies. To clearly outline the core characteristics and research priorities of existing studies, the key information from representative literature is summarized in Table 1 below.

Existing research on PB recycling primarily focuses on game theory analysis, channel optimization, and profit allocation in deterministic environments, with a particular emphasis on how external constraints—such as policy subsidies, incentive and penalty mechanisms, and carbon trading—influence the behavior of recycling entities. However, current research still suffers from the following shortcomings: First, most studies assume that DM information is entirely certain, failing to adequately account for the ambiguity, uncertainty, and subjective preferences of decision-makers inherent in the recycling DM process. Second, there is a lack of effective modeling methods and distance measures to address the robustness of recycling decisions under the interference of noisy information. Third, some research conclusions rely on specific policy scenarios and stakeholder structures, limiting their adaptability to the complex and dynamic real-world recycling environment. Therefore, there remains significant room for improvement in the actual DM process for PB recycling. There is an urgent need to develop a multi-attribute decision-making (MADM) method capable of effectively handling fuzzy information and resisting noise interference, in order to enhance the scientific rigor and robustness of recycling alternative selection. In solving practical DM problems, the real DM environment is often distinguished by fuzziness, uncertainty, and subjectivity. This requires decision-makers to select the optimal alternative using multi-attribute evaluation data. MADM methods support decision-makers in prioritizing alternatives according to their attribute evaluations, thereby enabling the optimal solution to be identified. Furthermore, to address the uncertainty inherent in real-world problems, Guong [11] proposed the picture fuzzy sets (PFSs) built upon the intuitionistic fuzzy sets (IFSs) introduced by Atanassov [12]. By introducing membership, neutrality, and non-membership degrees, PFSs better assists decision-makers in addressing uncertainty in complex evaluation scenarios. PFSs have been extensively employed to handle problems such as MADM and pattern recognition. Wang et al. [13] proposed a MADM method founded on regret theory and picture fuzzy (PF) LINMAP. Han [14] put forward a MADM method using PF entropy and PF weighted symmetric cross-entropy. Zhang [15] proposed a MADM method derived from the PF power Heronian mean operator. Li et al. [16] presented a PF MADM method founded on the projection method and cross-entropy. Wang et al. [17] introduced a MADM method using the PF power geometric Heronian mean operator and the PF weighted power geometric Heronian mean operator. Wang et al. [18] transformed PFSs into three-dimensional fuzzy sets and developed a PF inference algorithm with the least squares model. Zheng [19] improved the score function in the PF environment to solve MADM problems. Fan [20] et al. applied the TODIM method based on the CRITIC method and regret theory in the PF environment for green supplier selection. Zhu et al. [21] proposed two new dispersion measures for interval-valued PFSs based on Jensen–Sahnnon divergence and developed an enhanced TOPSIS method for interval-valued PFSs.

A classic MADM method known as the Technique for Order Preference by Similarity to ideal Solution (TOPSIS) was proposed by Hwang and Yoon [22] in 1981, offering advantages such as intuitiveness, simplicity, and flexibility. Zeng and Mu [23] proposed a TOPSIS DM method with hybrid weighted measures based on a novel Pythagorean fuzzy distance measure. Li et al. [24] proposed a dynamic triangular fuzzy MADM method founded on TOPSIS for MADM problems with triangular fuzzy attribute values. Wu et al. [25] developed an improved hesitant fuzzy linguistic distance measure. Su et al. [26] put forward a dynamic gray relational TOPSIS evaluation method based on globally improved normalization of weakening buffer operators to realize dynamic MADM. Jin et al. [27] proposed a TOPSIS DM method using multi-granular hesitant fuzzy linguistic term sets. Jiang et al. [28] presented an improved TOPSIS method founded on the Zhenyuan integral to comprehensively characterize the correlation among multiple attributes.

TOPSIS has been successfully adapted to fuzzy domains such as IFSs and PFSs, effectively addressing MADM problems under uncertainty. Singh et al. [29] designed a VIKOR-TOPSIS method using their proposed PF knowledge measure and accuracy measure. Sun [30] introduced PFSs into multi-criteria DM by combining the absolute positive ideal solution (PIS) and negative ideal solution (NIS) based on an improved TOPSIS method and an enhanced gray relational projection founded on the weighted Mahalanobis distance. Nurdan et al. [31] presented the PF-Z-AHP method and the PF-Z-TOPSIS method for ranking alternatives. Jin et al. [32] proposed a multi-criteria DM method applicable for the PF environment by combining the covering PF rough set model with the TOPSIS method. Dhumras et al. [33] integrated q-interval PFSs and two-parameter discriminant information measure theory into a TOPSIS-VIKOR DM model oriented toward federated learning to construct a DM framework for e-marketing strategy options.

As the core foundation for calculating the proximity of alternatives to the ideal solution in the TOPSIS method, the precision of distance measures directly determines the dependability of decision outputs. The various PF distance measures proposed in the academic literature [34,35,36,37,38,39,40,41,42,43,44,45,46,47,48,49,50,51,52] still have some shortcomings. The Hamming distance and Euclidean distance extended to PFSs by Guong [11] do not consider the reality that decision-makers place varying degrees of importance on membership, neutrality, and non-membership in actual DM scenarios, lack robustness, are sensitive to changes in attribute weights, and are ill-suited for complex DM problems characterized by significant expert disagreement and incomplete information. To overcome the limitations of existing distance measures mentioned above, this paper constructs a novel PF distance measure founded on the Bray–Curtis distance and combines it with the TOPSIS MADM method based on the novel PF distance measure. Finally, a case study on PB recycling confirms the feasibility and effectiveness of this method.

## 2. Preliminaries

### 2.1. Picture Fuzzy Set and Related Concepts

**Definition** **1** **(**[1]**).** *Let* X *be a universe of discourse. Then a PFS* G *on* X *can be defined as* $G\left(x\right)=\left\{\langle x,\xi_{G}\left(x\right),\psi_{G}\left(x\right),\vartheta_{G}\left(x\right)\left|x\in X\right.\rangle \right\}$*, where* $\xi_{G}\left(x\right)$ *denotes the membership degree of* x *to* G*,* $\psi_{G}\left(x\right)$ *denotes the neutrality degree of* x *to* G*, and* $\vartheta_{G}\left(x\right)$ *denotes the non-membership degree of* x *to* G*.* $\xi_{G}\left(x\right)$*,* $\psi_{G}\left(x\right)$ *and* $\vartheta_{G}\left(x\right)$ *all* $\in \left[0,1\right]$*.* $\xi_{G}\left(x\right)$*,* $\psi_{G}\left(x\right)$ *and* $\vartheta_{G}\left(x\right)$ *satisfy* $0\leq \xi_{G}\left(x\right)+\psi_{G}\left(x\right)+\vartheta_{G}\left(x\right)\geq 1$ *and* ∀x∈X*. The refusal degree of* X *when* x∈X *is* $\pi_{G}\left(x\right)=1-\left(\xi_{G}\left(x\right)+\psi_{G}\left(x\right)+\vartheta_{G}\left(x\right)\right)$*.*

**Definition** **2** **(**[12]**).** *Let* $\alpha =\left(\xi_{\alpha },\psi_{\alpha },\vartheta_{\alpha }\right)$ *and* $\beta =\left(\xi_{\beta },\psi_{\beta },\vartheta_{\beta }\right)$ *be two PF numbers, then the algorithm between them is as follows:*

*(1)*  $\alpha ⊕\beta =\left(\xi_{\alpha }+\xi_{\beta }-\xi_{\alpha }\xi_{\beta },\psi_{\alpha }\psi_{\beta },\vartheta_{\alpha }\vartheta_{\beta }\right)$*;*
*(2)*  $\alpha ⊗\beta =\left(\xi_{\alpha }\xi_{\beta },\psi_{\alpha }+\psi_{\beta }-\psi_{\alpha }\psi_{\beta },\vartheta_{\alpha }+\vartheta_{\beta }-\vartheta_{\alpha }\vartheta_{\beta }\right)$*;*
*(3)*  $\lambda \alpha =\left(1-{\left(1-\xi_{\alpha }\right)}^{\lambda },{\psi_{\alpha }}^{\lambda },{\vartheta_{\alpha }}^{\lambda }\right)$*;*
*(4)*  $\alpha^{\lambda }=\left({\xi_{\alpha }}^{\lambda },1-{\left(1-\psi_{\alpha }\right)}^{\lambda },1-{\left(1-\vartheta_{\alpha }\right)}^{\lambda }\right),\left(\lambda >0\right)$.

**Definition** **3.** *Let* $ℑ=\left\{\left(x_{i},\xi_{ℑ}\left(x_{i}\right),\psi_{ℑ}\left(x_{i}\right),\vartheta_{ℑ}\left(x_{i}\right)\right)\left|x_{i}\in X\right.\right\}$ *and* $ℜ=\left\{\left(x_{i},\xi_{ℜ}\left(x_{i}\right),\psi_{ℜ}\left(x_{i}\right),\vartheta_{ℜ}\left(x_{i}\right)\right)\left|x_{i}\in X\right.\right\}$ *be two PFSs on* $X=\left\{x_{1},x_{2},\cdots ,x_{n}\right\}$*. Part of the existing PFS distance measures are shown as Table 2.*

### 2.2. A Novel Picture Fuzzy Distance

The Bray–Curtis distance measures the similarity between coordinates primarily used in ecology and environmental science [55]. It takes values in the interval [0, 1] and can also be used to calculate the difference between coordinates. In an n-dimensional space, the Bray–Curtis distance is

(1) $$d\left(p,q\right)=\frac{\sum_{i=1}^{n}\left|p_{i}-q_{i}\right|}{\sum_{i=1}^{n}p_{i}+\sum_{i=1}^{n}q_{i}}.$$

The Bray–Curtis distance offers several advantages, including resistance to outliers, computational simplicity, high interpretability, and the absence of the need for complex normalization preprocessing. These characteristics are preserved when extended to the PF distance measure, which adapts to the fuzziness of expert ratings. It mitigates the interference of extreme scores in a single dimension on the overall distance calculation and overcomes the limitations of traditional fuzzy distances-such as Euclidean and Manhattan distances which focus solely on the two-dimensional information of membership and non-membership. It accurately captures neutral attitudes in expert evaluations, making the distance metric more aligned with the real-world DM scenarios.

**Definition** **4.** *Let* $ℑ=\left\{\left(x_{i},\xi_{ℑ}\left(x_{i}\right),\psi_{ℑ}\left(x_{i}\right),\vartheta_{ℑ}\left(x_{i}\right)\right)\left|x_{i}\in X\right.\right\}$ *and* $ℜ=\left\{\left(x_{i},\xi_{ℜ}\left(x_{i}\right),\psi_{ℜ}\left(x_{i}\right),\vartheta_{ℜ}\left(x_{i}\right)\right)\left|x_{i}\in X\right.\right\}$ *be two PFSs on* $X=\left\{x_{1},x_{2},\cdots ,x_{n}\right\}$ *, then the following expression* 

(2) $$d\left(ℑ,ℜ\right)=\frac{\sum_{i=1}^{n}\left[\rho_{1}\left|\xi_{ℑ}\left(x_{i}\right)-\xi_{ℜ}\left(x_{i}\right)\right|+\rho_{2}\left|\psi_{ℑ}\left(x_{i}\right)-\psi_{ℜ}\left(x_{i}\right)\right|+\rho_{3}\left|\vartheta_{ℑ}\left(x_{i}\right)-\vartheta_{ℜ}\left(x_{i}\right)\right|\right]}{\sum_{i=1}^{n}\left(\xi_{ℑ}\left(x_{i}\right)+\psi_{ℑ}\left(x_{i}\right)+\vartheta_{ℑ}\left(x_{i}\right)\right)+\sum_{i=1}^{n}\left(\xi_{ℜ}\left(x_{i}\right)+\psi_{ℜ}\left(x_{i}\right)+\vartheta_{ℜ}\left(x_{i}\right)\right)+\varepsilon }$$

 *is defined as the PF distance measure of* ℑ *and* ℜ*, where* $\sum_{d=1}^{3}\rho_{d}=1$ *and* ε=0.001*.*

The distance measure defined in this paper is a semimetric that satisfies non-negativity and symmetry but does not satisfy the triangle inequality. The Bray–Curtis distance focuses on analyzing the proportion of differences within the “belonging-non- belonging-neutral” ternary structure of PFSs and offers certain advantages in fuzzy DM and the quantification of compositional differences.

**Theorem** **1.** *The PF distance* *measure* $d\left(ℑ,ℜ\right)$ *satisfies the two basic axioms of distance metric:*

*(1)*  *Non-negativity: For any PFSs* ℑ *and* ℜ*,* $d\left(ℑ,ℜ\right)\geq 0$*, and* $d\left(ℑ,ℜ\right)=0$ *if and only of* ℑ=ℜ*.*
*(2)*  *Symmetry: For any PFSs* ℑ *and* ℜ*,* $d\left(ℑ,ℜ\right)=d\left(ℜ,ℑ\right)$*.*

**Proof.**  (1)

$$\sum_{i=1}^{n}\left[\rho_{1}\left|\xi_{ℑ}\left(x_{i}\right)-\xi_{ℜ}\left(x_{i}\right)\right|+\rho_{2}\left|\psi_{ℑ}\left(x_{i}\right)-\psi_{ℜ}\left(x_{i}\right)\right|+\rho_{3}\left|\vartheta_{ℑ}\left(x_{i}\right)-\vartheta_{ℜ}\left(x_{i}\right)\right|\right]\geq 0$$

and

$$\sum_{i=1}^{n}\left(\xi_{ℑ}\left(x_{i}\right)+\psi_{ℑ}\left(x_{i}\right)+\vartheta_{ℑ}\left(x_{i}\right)\right)+\sum_{i=1}^{n}\left(\xi_{ℜ}\left(x_{i}\right)+\psi_{ℜ}\left(x_{i}\right)+\vartheta_{ℜ}\left(x_{i}\right)\right)+\varepsilon >0,$$

thus $d\left(ℑ,ℜ\right)\geq 0$.

(2) Absolute value has symmetry, that is, $\left|\xi_{ℑ}\left(x_{i}\right)-\xi_{ℜ}\left(x_{i}\right)\right|=\left|\xi_{ℜ}\left(x_{i}\right)-\xi_{ℑ}\left(x_{i}\right)\right|$. The same applies to other terms, and the denominator symmetric. Hence $d\left(ℑ,ℜ\right)=d\left(ℜ,ℑ\right)$. □

**Corollary** **1.** *For any two PFSs* $ℑ=\left\{\left(x_{i},\xi_{ℑ}\left(x_{i}\right),\psi_{ℑ}\left(x_{i}\right),\vartheta_{ℑ}\left(x_{i}\right)\right)\left|x_{i}\in X\right.\right\}$ *and* $ℜ=\left\{\left(x_{i},\xi_{ℜ}\left(x_{i}\right),\psi_{ℜ}\left(x_{i}\right),\vartheta_{ℜ}\left(x_{i}\right)\right)\left|x_{i}\in X\right.\right\}$*, their distance measure* $d\left(ℑ,ℜ\right)$ *satisfies* $0\leq d\left(ℑ,ℜ\right)<1$*.*

**Proof.**  Terms $\rho_{1}\left|\xi_{ℑ}\left(x_{i}\right)-\xi_{ℜ}\left(x_{i}\right)\right|\geq 0$, $\rho_{2}\left|\psi_{ℑ}\left(x_{i}\right)-\psi_{ℜ}\left(x_{i}\right)\right|\geq 0$, $\rho_{3}\left|\vartheta_{ℑ}\left(x_{i}\right)-\vartheta_{ℜ}\left(x_{i}\right)\right|\geq 0$ and denominator are positive, hence $d\left(ℑ,ℜ\right)\geq 0$. For any non-negative real numbers s and t, we have $\left|s-t\right|\leq s+t$. Because $\rho_{1},\rho_{2},\rho_{3}\geq 0$ and $\sum_{d=1}^{3}\rho_{d}=1$, each $\rho_{d}\leq 1$. Therefore,

$$\rho_{1}\left|\xi_{ℑ}\left(x_{i}\right)-\xi_{ℜ}\left(x_{i}\right)\right|\leq \rho_{1}\left(\xi_{ℑ}\left(x_{i}\right)+\xi_{ℜ}\left(x_{i}\right)\right)\leq \xi_{ℑ}\left(x_{i}\right)+\xi_{ℜ}\left(x_{i}\right),$$

$$\rho_{2}\left|\psi_{ℑ}\left(x_{i}\right)-\psi_{ℜ}\left(x_{i}\right)\right|\leq \psi_{ℑ}\left(x_{i}\right)+\psi_{ℜ}\left(x_{i}\right),$$

$$\rho_{2}\left|\vartheta_{ℑ}\left(x_{i}\right)-\vartheta_{ℜ}\left(x_{i}\right)\right|\leq \vartheta_{ℑ}\left(x_{i}\right)+\vartheta_{ℜ}\left(x_{i}\right).$$

Summing over i=1,2,…,n:

$$\begin{array}{l} \sum_{i=1}^{n}\left(\rho_{1}\left|\xi_{ℑ}\left(x_{i}\right)-\xi_{ℜ}\left(x_{i}\right)\right|+\rho_{2}\left|\psi_{ℑ}\left(x_{i}\right)-\psi_{ℜ}\left(x_{i}\right)\right|+\rho_{3}\left|\vartheta_{ℑ}\left(x_{i}\right)-\vartheta_{ℜ}\left(x_{i}\right)\right|\right)\\ \leq \sum_{i=1}^{n}\left(\xi_{ℑ}\left(x_{i}\right)+\xi_{ℜ}\left(x_{i}\right)+\psi_{ℑ}\left(x_{i}\right)+\psi_{ℜ}\left(x_{i}\right)+\vartheta_{ℑ}\left(x_{i}\right)+\vartheta_{ℜ}\left(x_{i}\right)\right). \end{array}$$

Denote

$$S=\sum_{i=1}^{n}\left(\xi_{ℑ}\left(x_{i}\right)+\xi_{ℜ}\left(x_{i}\right)+\psi_{ℑ}\left(x_{i}\right)+\psi_{ℜ}\left(x_{i}\right)+\vartheta_{ℑ}\left(x_{i}\right)+\vartheta_{ℜ}\left(x_{i}\right)\right)\geq 0.$$

Then the numerator

$$S=\sum_{i=1}^{n}\left(\xi_{ℑ}\left(x_{i}\right)+\xi_{ℜ}\left(x_{i}\right)+\psi_{ℑ}\left(x_{i}\right)+\psi_{ℜ}\left(x_{i}\right)+\vartheta_{ℑ}\left(x_{i}\right)+\vartheta_{ℜ}\left(x_{i}\right)\right)\geq 0,$$

$$\sum_{i=1}^{n}\left[\rho_{1}\left|\xi_{ℑ}\left(x_{i}\right)-\xi_{ℜ}\left(x_{i}\right)\right|+\rho_{2}\left|\psi_{ℑ}\left(x_{i}\right)-\psi_{ℜ}\left(x_{i}\right)\right|+\rho_{3}\left|\vartheta_{ℑ}\left(x_{i}\right)-\vartheta_{ℜ}\left(x_{i}\right)\right|\right]\leq S,$$

$$\sum_{i=1}^{n}\left(\xi_{ℑ}\left(x_{i}\right)+\psi_{ℑ}\left(x_{i}\right)+\vartheta_{ℑ}\left(x_{i}\right)\right)+\sum_{i=1}^{n}\left(\xi_{ℜ}\left(x_{i}\right)+\psi_{ℜ}\left(x_{i}\right)+\vartheta_{ℜ}\left(x_{i}\right)\right)+\varepsilon =S+\varepsilon$$

with ε>0. Hence $d\left(ℑ,ℜ\right)\leq \frac{S}{S+\varepsilon }<1$. We can obtain $0\leq d\left(ℑ,ℜ\right)<1$. □

**Remark** **1.** *If* ε=0 *, we could only conclude* $d\left(ℑ,ℜ\right)\leq 1$ *, and equality may hold. However, the definition fixes* ε=0.001 *, so the strict inequality* <1 *is guaranteed.*

**Corollary** **2.** *Let* $d\left(ℑ,ℜ\right)$ *be the distance* *measure between two PFSs* ℑ *and* ℜ *. If the similarity degree is defined as* $s\left(ℑ,ℜ\right)=1-d\left(ℑ,ℜ\right)$ *, then* $s\left(ℑ,ℜ\right)$ *satisfies the following properties:*

*(1)*  $0<s\left(ℑ,ℜ\right)\leq 1$*;*
*(2)*  $s\left(ℑ,ℜ\right)=1$*, if and only* ℑ=ℜ *;*
*(3)*  $s\left(ℑ,ℜ\right)=s\left(ℜ,ℑ\right)$.

**Proof.**  (1) From the boundedness of $d\left(ℑ,ℜ\right)$, we know $0\leq d\left(ℑ,ℜ\right)<1$. Multiplying both sides of the inequality by −1 gives $-1<-d\left(ℑ,ℜ\right)\leq 0$. It follows that $0<1-d\left(ℑ,ℜ\right)\leq 1$, that is, $0<s\left(ℑ,ℜ\right)\leq 1$.

(2) Sufficiency: If ℑ=ℜ, then $d\left(ℑ,ℜ\right)=0$ by the identity property, so $s\left(ℑ,ℜ\right)=1-d\left(ℑ,ℜ\right)=1$. Necessity: If $s\left(ℑ,ℜ\right)=1$, then $d\left(ℑ,ℜ\right)=0$. By the identify property, $d\left(ℑ,ℜ\right)=0$ if and only if ℑ=ℜ.
(3) From the symmetry of $d\left(ℑ,ℜ\right)$, we have $d\left(ℑ,ℜ\right)=d\left(ℜ,ℑ\right)$. Therefore, $s\left(ℑ,ℜ\right)=1-d\left(ℑ,ℜ\right)=1-d\left(ℜ,ℑ\right)=s\left(ℜ,ℑ\right)$. □

Next, the implications of different parameter values $\rho_{d}$ in the newly proposed PF distance measure.

The weighting parameter $\rho_{d}$ in $d\left(ℑ,ℜ\right)$ incorporates decision-makers preferences and scenario constrains into the fuzzy information measure. Essentially, it assigns values to the relative importance of the three dimensions—membership, neutrality, and non-membership—thereby making the fuzzy distance measure suitable for different DM needs. The following presents four typical weighting configurations and their corresponding practical implications.

**Scenario 1** Balanced weighting, with typical weights sets as $\rho_{1}=\rho_{2}=\rho_{3}$.

Under this configuration, the differences across the three dimensions—membership, neutrality, and non-membership—are assigned equal weights, treating each element’s differences in positive, neutral, and negative information on an equal basis. This approach is suitable for scenarios where there are no prior DM preferences and is used for unbiased preliminary comparisons of options and overall assessments of differences.

**Scenario 2** Positive-difference priority type, with weights sets as $\rho_{1}=0.8$, $\rho_{2}=0.1$, $\rho_{3}=0.1$.

In this scenario, membership differences become the primary determining factor in distance measures, while the influence of moderate strength and non-membership is significantly reduced. This approach is suitable for positive, goal-oriented DM, focusing on the consistency and variation in positive indicators such as core consensus and satisfaction, and enabling rapid identification of the relative strengths and weakness of solutions along positive value dimensions.

**Scenario 3** Negative-difference priority type, with weights sets as $\rho_{1}=0.1$, $\rho_{2}=0.1$, $\rho_{3}=0.8$.

Non-membership differences are the dominant dimension in this scenario, while differences in membership and moderate-strength dimensions serve only as supplementary factors. This approach is suitable for DM that prioritizes negative constraints, with negative indicators such as risk level, compliance, and failure risk being the primary focus. This configuration enhances sensitivity to negative risks, enabling the precise identification of high-risk solutions.

**Scenario 4** Neutral-difference priority type, with weights sets as $\rho_{1}=0.1$, $\rho_{2}=0.8$, $\rho_{3}=0.1$.

Differences in neutrality are significantly amplified, making it suitable for DM scenarios sensitive to indecision. It focuses on characterizing the degree of indecision or the uncertainty of ambiguous information within the subject of study, enabling the precise capture of changes in neutrality and enhancing the DM process’s ability to perceive ambiguity and indecision.

### 2.3. Comparative Analysis of Picture Fuzzy Distance

In practical applications of MADM, evaluation information is prone to minor disturbances caused by measurement errors, subjective cognitive biases, and external environmental interference. Therefore, the robustness of a distance measure—its ability to withstand interference from anomalous or noisy data—is a key indicator for assessing the overall performance of PFS distance measures. The higher the robustness, the smaller the fluctuation in distance calculation results under minor data disturbances, indicating that the decision model’s output is more stable and reliable, making it more suitable for decision analysis in complex and uncertain environments.

To examine the proposed PF distance measure’s robustness against noisy data, this paper conducts a noise sensitivity comparative analysis between the proposed distance and the five aforementioned PF distance measures. By introducing different levels of noise into the test data, the stability performance of the distance measures under noise interference is quantitatively evaluated. Table 3 presents the robustness metrics of the PFS distance measures under noise disturbance.

**Figure 1:**
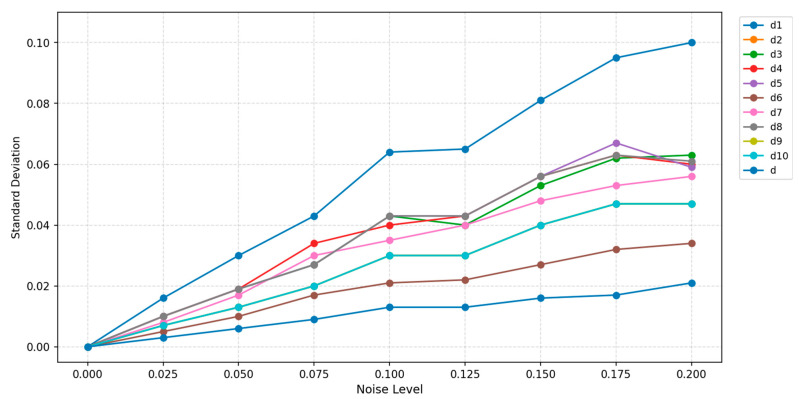
Noise sensitivity of distance measures.

This study employs a “noise intensity–standard deviation” curve to conduct a comparative analysis of the robustness of 11 PFS distance measures. The horizontal axis represents noise intensity, which characterizes the severity of data disturbance; the vertical axis represents the standard deviation of the distance calculation results, which characterizes the level of fluctuation of the measure under disturbance. The smaller the slope of the standard deviation as noise intensity increases, the lower the sensitivity of the distance measure to data perturbations, and the stronger its robustness. Combined with the analysis results in Figure 1, the curve shows that as in noise level increases, the slope of the PF distance measure proposed above is significantly lower than those of the existing measures. This demonstrates that the proposed distance can effectively resist noise interference, is applicable to practical DM scenarios with complex noise and high uncertainty, and can more accurately reflect the real differences between PFSs.

## 3. TOPSIS Multi-Attribute Decision-Making Based on the Novel Distance Measure

### Specific Steps for TOPSIS Multi-Attribute Decision-Making

The specifics of the improved TOPSIS MADM method derived from the novel PF distance are as follows. For the MADM problem under the PF environment, let $A_{i}$ (i=1,2,⋯m) be the alternatives, $C_{j}$ (j=1,2,⋯n) be the evaluation criterion, and $w_{j}$ (j=1,2,⋯n) be the corresponding weight of the attribute, where $0\leq w_{j}\leq 1$ and $\sum_{j=1}^{n}w_{j}=1$. $x_{ij}=\left(\xi_{ij},\psi_{ij},\vartheta_{ij}\right)$ denotes the PF number of alternative $A_{i}$ with respect to evaluation criterion $C_{j}$, $\xi_{ij}\geq 0$, $\psi_{ij}\geq 0$, $\vartheta_{ij}\geq 0$ and $\xi_{ij}+\psi_{ij}+\vartheta_{ij}\leq 1$.

**Step 1** Construct the PF decision matrix $X={\left(x_{ij}\right)}_{m\times n}$.

**Step 2** Normalize the PF decision matrix. Due to different types of attributes, it is necessary to normalize the matrix. Let $z_{ij}=x_{ij}=\left(\xi_{ij},\psi_{ij},\vartheta_{ij}\right)$ denote the benefit attribute and ${\tilde{z}}_{ij}=x_{ij}=\left(\xi_{ij},\psi_{ij},\vartheta_{ij}\right)$ denote the cost attribute, to obtain the normalized matrix $Z={\left(z_{ij}\right)}_{m\times n}$.

**Step 3** Determine the positive PIS and the NIS.

For each attribute j, let the PIS be $A^{+}=\left(v_{1}^{+},v_{2}^{+},\ldots ,v_{n}^{+}\right)$, where

(3) $$v_{j}^{+}=\left(\max\xi_{ij},\min\psi_{ij},\min\vartheta_{ij}\right).$$

For each attribute j, let the NIS be $A^{-}=\left(v_{1}^{-},v_{2}^{-},\ldots ,v_{n}^{-}\right)$, where

(4) $$v_{j}^{-}=\left(\min\xi_{ij},\max\psi_{ij},\max\vartheta_{ij}\right).$$

**Step 4** Calculate the distance between each alternative and the PIS, as well as the NIS.

Distance to the PIS:

(5) $$d_{ij}^{+}=d\left(z_{ij},v_{j}^{+}\right)=\frac{\rho_{1}\left|\xi_{ij}-\xi_{j}^{+}\right|+\rho_{2}\left|\psi_{ij}-\psi_{j}^{+}\right|+\rho_{3}\left|\vartheta_{ij}-\vartheta_{j}^{+}\right|}{\left(\xi_{ij}+\psi_{ij}+\vartheta_{ij}\right)+\left(\xi_{j}^{+}+\psi_{j}^{+}+\vartheta_{j}^{+}\right)+\varepsilon }.$$

Distance to the NIS:

(6) $$d_{ij}^{-}=d\left(z_{ij},v_{j}^{-}\right)=\frac{\rho_{1}\left|\xi_{ij}-\xi_{j}^{-}\right|+\rho_{2}\left|\psi_{ij}-\psi_{j}^{-}\right|+\rho_{3}\left|\vartheta_{ij}-\vartheta_{j}^{-}\right|}{\left(\xi_{ij}+\psi_{ij}+\vartheta_{ij}\right)+\left(\xi_{j}^{-}+\psi_{j}^{-}+\vartheta_{j}^{-}\right)+\varepsilon }.$$

**Step 5** The weighted distance to the PIS is $D_{i}^{+}=\sum_{j=1}^{n}w_{j}\cdot d_{ij}^{+}$, and the weighted distance to the NIS is $D_{i}^{-}=\sum_{j=1}^{n}w_{j}\cdot d_{ij}^{-}$.

**Step 6** Obtain relative proximity and rank the alternative solutions. Relative proximity $C_{i}$ is used to measure how close a solution is to the optimal solution. It is calculated using the formula

(7) $$RC_{i}=\frac{D_{i}^{-}}{D_{i}^{+}+D_{i}^{-}},$$

where $RC_{i}\in \left[0,1\right]$. The relative proximity closer to 1 indicates a solution nearer to the optimal solution. Ranking the $RC_{i}$ values of each solution from highest to lowest to determine the order of preference among the solutions.

## 4. Case Study

An EV manufacturer, aiming to achieve its carbon strategy, complete the closed-loop industrial chain, and reduce recycling costs and environmental risks, plans to establish a standardized PB recycling system. To this end, the company needs to select the optimal solution from four mainstream recycling models currently prevalent in the industry. It faces multiple challenges in the DM process. Firstly, the assessment of recycling models involves multi-dimensional attributes such as economics, carbon reduction, environmental protection, technology and policy, with interdependent relationships existing between these attributes. Secondly, the evaluation data exhibits significant ambiguity and neutrality. Due to rapid advancements in recycling technology and dynamic adjustments in policy directions, industry experts find it difficult to express clear ‘support’ or ‘opposition’ towards each model, resulting in ambiguous judgements such as ‘abstention’ or ‘neutrality’. Thirdly, the relative importance of different evaluation attributes varies, necessitating the allocation of appropriate weights to reflect the priorities of the DM process. Given this situation, the DM problem at hand constitutes a fuzzy MADM problem. PFSs can effectively address the shortcomings of traditional intuitionistic fuzzy sets; when combined with the improved novel PF distance measure, they can further enhance the resolution of fuzzy information and DM accuracy. Consequently, this paper employs a TOPSIS MADM method utilizing the novel PF distance measure to optimally select from the four PB recycling models, thereby providing theoretical support and practical guidance for enterprises to formulate scientific recycling strategies.

**Figure 2:**
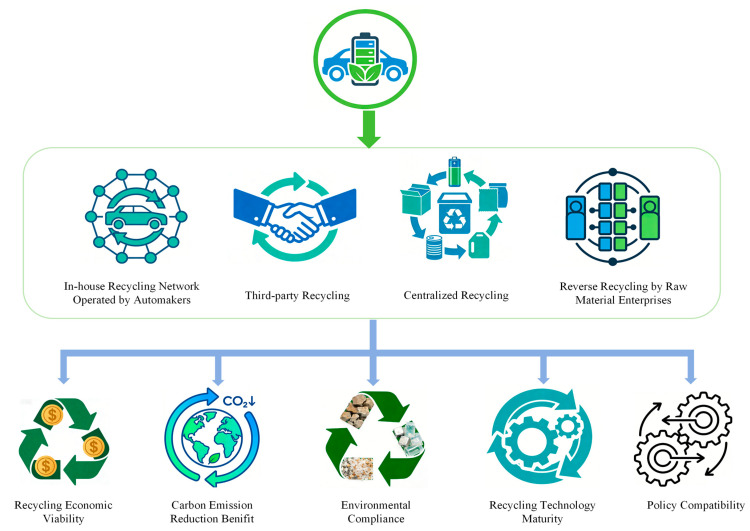
Hierarchical diagram of MADM for PB recycling modes.

To scientifically evaluate the overall performance of PB recycling models, this paper draws on strategic objectives and industry practices, and, with reference to Huang et al. [56], constructs an evaluation framework comprising five key dimensions and four recycling models. Let the evaluation attribute for an EV manufacturer’s PB recycling model be represented by the set C, where $C_{1}$ denotes economic viability of recycling, $C_{2}$ denotes carbon emission reduction benefits, $C_{3}$ denotes environmental compliance, $C_{4}$ denotes technical maturity of recycling, and $C_{5}$ denotes policy alignment. Set A represents four corporate PB recycling models, $A=\left\{A_{1},A_{2},A_{3},A_{4}\right\}$. $A_{1}$ denotes an in-house recycling network operated by the EV manufacturer. $A_{2}$ denotes collaboration with a third-party specialist recycling company. $A_{3}$ denotes a centralized recycling mode. and $A_{4}$ denotes reverse recycling by raw material suppliers. And Figure 2 is the hierarchical diagram of MADM for PB recycling modes.

Five EV manufacturers and research experts in the field of PB recycling were invited to independently rate the importance of each evaluation attribute using a Likert scale. The average of the experts’ ratings was calculated, and a normalization method was used to determine the weight of each criterion, thereby deriving the attribute weights $w=\left(0.22,0.25,0.18,0.20,0.15\right)$. The weight parameters $\rho_{d}$ in $d\left(ℑ,ℜ\right)$ are set to $\rho_{1}=\rho_{2}=\rho_{3}$. By evaluating the various attributes of the four recycling modes, the initial decision matrix is obtained as follows

$$Z=\left(\begin{array}{ccccc} \left[0.63,0.21,0.14\right] & \left[0.55,0.29,0.12\right] & \left[0.78,0.12,0.09\right] & \left[0.64,0.18,0.12\right] & \left[0.57,0.17,0.20\right]\\ \left[0.77,0.12,0.10\right] & \left[0.69,0.18,0.09\right] & \left[0.70,0.18,0.11\right] & \left[0.56,0.29,0.12\right] & \left[0.63,0.15,0.11\right]\\ \left[0.63,0.18,0.12\right] & \left[0.76,0.12,0.09\right] & \left[0.79,0.09,0.10\right] & \left[0.81,0.09,0.09\right] & \left[0.74,0.12,0.09\right]\\ \left[0.69,0.18,0.10\right] & \left[0.65,0.22,011\right] & \left[0.57,0.28,0.12\right] & \left[0.65,0.21,0.11\right] & \left[0.48,0.31,0.18\right] \end{array}\right)$$

### 4.1. Solution Procedure

**Step 1** The five evaluation attributes selected in this paper are all positive indicators, so no conversion from cost-type to benefit-type attributes is required. Thus, the standardized matrix is consistent with the original PF decision matrix presented in Table 4.

**Step 2** Calculate the PISs and NISs using the formulas (3) and (4), as displayed in Table 5.

**Step 3** Calculate the distances from each alternative to the PISs and NISs. Using the novel PF distance measure formulas (5) and (6), the distances from each alternative to the PISs and NISs for each criterion are calculated, as shown in Table 6 and Table 7.

**Step 4** Calculate the weighted distances. For each alternative, the distances to the PISs and NISs are weighted and aggregated according to the attribute weights, resulting in the weighted total distances to the PIS and NIS, respectively. Weighted total distances of the four alternatives are shown in Table 8.

**Step 5** Calculate the relative proximity and rank the alternatives. The relative proximity is calculated according to the formula (7), which measures the proximity of each alternative to the PIS.

$$RC_{1}=0.3423,\;RC_{2}=0.5536,\;RC_{3}=0.8796,\;RC_{4}=0.2673.$$

Based on the results of the relative proximity calculations outlined above, it can be concluded that Alternative 3, the centralized recycling model, is the optimal solution.

### 4.2. Validity Analysis

To validate the effectiveness of the proposed PF distance measure and the method presented herein, the aforementioned PF distance measures are applied to the same case study for comparative analysis. The decision results of each distance are presented in Table 9.

As demonstrated in Table 9, the proposed PF distance measure gives ranking results for the optimal alternative that are consistent compared with the Hamming distance, the Euclidean distance, and the distance proposed by Dinh and Thao, which demonstrates the effectiveness of the MADM method based on the novel PF distance measure proposed in this paper.

### 4.3. Parameter Sensitivity Analysis

#### 4.3.1. Sensitivity Analysis of Attribute Weights

**Figure 3:**
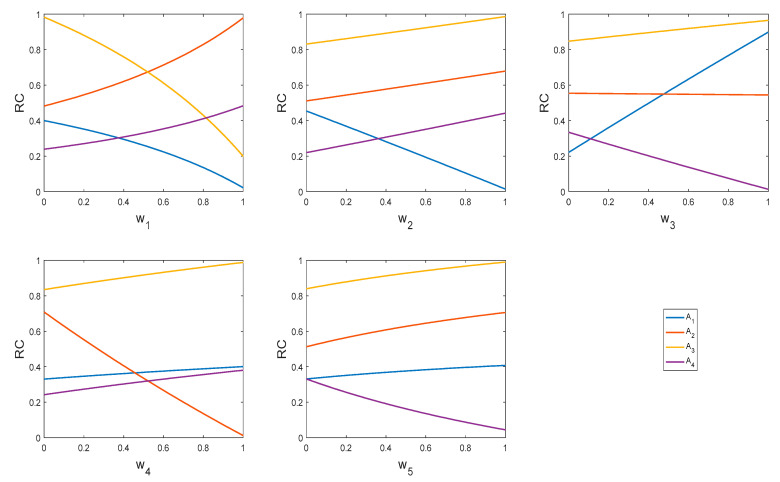
Curves of the relative proximity $RC_{i}$ of each alternative with different attribute weights.

To further test the stability and reliability of the proposed DM model and prevent decision mistakes arising from the subjectivity of attribute weight settings, a sensitivity analysis of attribute weights is conducted. By changing the weight j of attribute $w_{j}$ and re-normalizing the remaining four attribute weights according to their original proportions, the relative proximity $RC_{i}$ of each alternative under the new weight combination is calculated. The curves of $RC_{i}$ as a function of $w_{j}$ are displayed in Figure 3.

From Figure 3, it can be seen that for $A_{3}$, its relative proximity is significantly higher than those of the other alternatives across the entire range of weight variations, with only a slight decrease when a few attribute weights take extreme values. For $A_{2}$, its relative proximity is stable between 0.5 and 0.6, and increases when the weight of $C_{1}$ or $C_{2}$ becomes lager, but it never exceeds that of $A_{3}$. The relative proximity of $A_{1}$ and $A_{4}$ is consistently below 0.4. Even when a certain attribute weight takes an extreme value, the ranking result of $A_{3}$ remains unchanged, indicating that $A_{3}$ does not depend on any specific attribute.

#### 4.3.2. Sensitivity Analysis of Picture Fuzzy Distance Measure Weights

**Figure 4:**
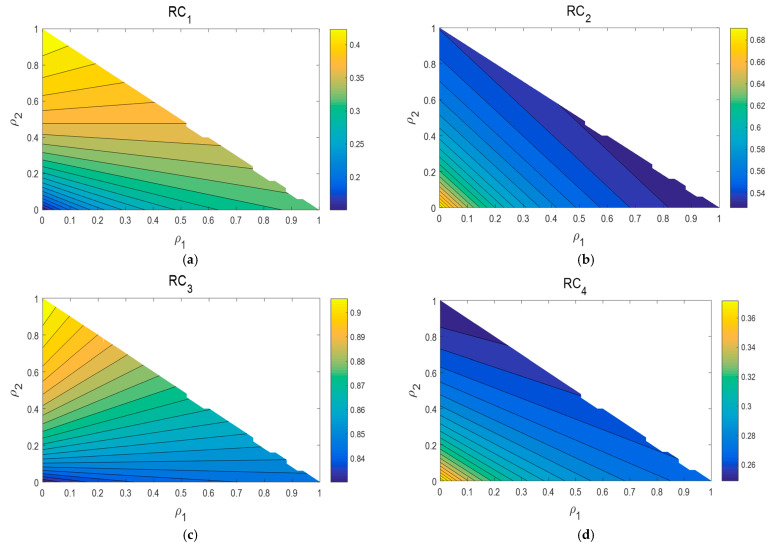
(**a**) Contour plot of the function of weights $\rho_{1}$ and $\rho_{2}$ for $RC_{1}$; (**b**) Contour plot of the function of weights $\rho_{1}$ and $\rho_{2}$ for $RC_{2}$; (**c**) Contour plot of the function of weights $\rho_{1}$ and $\rho_{2}$ for $RC_{3}$; (**d**) Contour plot of the function of weights $\rho_{1}$ and $\rho_{2}$ for $RC_{4}$.

To avoid the influence of weight settings for membership, neutrality, and non-membership on the decision results, this section performs a sensitivity analysis regarding the weights of the PF distance Measure. The weights $\rho_{1}$, $\rho_{2}$ and $\rho_{3}$ for membership, neutrality, and non-membership in the PF distance measure $d\left(ℑ,ℜ\right)$ satisfy $\sum_{d=1}^{3}\rho_{d}=1$. Let $\rho_{1}$ and $\rho_{2}$ vary within [0, 1] with a step size of 0.02, and $\rho_{3}=1-\rho_{1}-\rho_{2}$. For each pair $\left(\rho_{1},\rho_{2}\right)$, all distances are recalculated, and the relative proximity RC for each alternative is calculated, from which the optimal alternative is identified. Figure 4 shows the variation trend of RC for each alternative as a function of ρ.

It can be seen from Figure 4 that for $A_{3}$, its $RC_{3}$ exceeds 0.85 in most regions, with sparse and flat contours, indicating its advantage in terms of stability. For $A_{2}$, its $RC_{2}$ approaches 0.6 when $\rho_{3}$ is large, and is slightly lower in other regions. The $RC_{4}$ values of $A_{1}$ and $A_{4}$ are always below 0.4. The ranking results are barely influenced by alterations in the distance weights. $A_{3}$ remains the optimal under all reasonable weight allocations.

Through the dual sensitivity analysis of attribute weights and distance weights, it is confirmed that $A_{3}$ has extremely strong robustness, as its relative proximity remains the highest no matter how the weight changes within a reasonable range.

## 5. Conclusions

This paper investigates MADM problems under a PF environment. A novel PF distance measure based on the Bray–Curtis distance is introduced, and a MADM method is constructed by combining it with the TOPSIS approach. Through theoretical analysis and a case study validation, the following conclusions are drawn:

(1) The newly proposed PF distance measure exhibits excellent robustness. It not only maintains good stability in scenarios where fuzzy numbers are contaminated by noise, but it also accounts for reflecting the decision-makers’ varying preferences for the three dimensions: membership, neutrality and non-membership degrees. A noise sensitivity comparison test was performed between the proposed distance and five typical existing PF distance measures. The distance measure proposed in this paper demonstrated the best robustness and exhibited greater flexibility and applicability compared to existing methods.
(2) The novel PF distance measure proposed herein was incorporated into the TOPSIS method, thereby establishing a new MADM model under the PF environment. Taking PB recycling for EVs as a practical example, the decision model was applied to select the optimal scheme. The results indicate that centralized recycling is the optimal decision. The ranking results from the TOPSIS method based on the proposed distance align with those of the other five existing PF distance measures, verifying the effectiveness of the decision model.
(3) Sensitivity analyses were performed separately on the attribute weights and the dimension weights of the PF distance measure. The results corroborate that the optimal scheme has good stability, further attesting to the reliability and practicality of the proposed model in real-world DM.

In summary, the new PF distance measure and the TOPSIS MADM model proposed in this paper effectively solve the core problems of noisy information and MADM under a PF environment. They provide a more robust and flexible novel method for the field of uncertain DM that is broadly applicable to various practical decision scenarios involving fuzziness and uncertainty. However, this research only verifies the effectiveness of the model based on the EV PB-recycling case, and its applicability to more complex, high-dimensional and big-data samples remains to be determined. In the future, we aim to introduce intelligent optimization algorithms to further enhance the flexibility and universality of the distance measures and improve the method’s ability to adapt to extremely ambiguous DM information.

## Figures and Tables

**Table 1:** Summary of literature on the PB recycling supply chain.

Literature	Supply Chain Stakeholders	Research Background	Core Research Content
Wang et al. [1]	PB manufacturers, EV manufacturers	No government intervention and subsides	An evolutionary game analysis of DM conditions and optimal strategies for corporate used battery recycling
Hu et al. [2]	Brand PB manufacturers, EV manufacturers, Third-party recyclers	Government subsidy policy	Analysis of the impact of subsidies on optimal strategies and profits in a closed-loop supply chain based on a single-channel expansion and dual-channel recycling model
Tang et al. [3]	Multi-stakeholder recycling supply chain	Government incentive and disciplinary policies	Compare the optimal operational decisions of supply chain members under six recycling modes
Ding et al. [4]	PB manufacturers, EV manufacturers, PB recyclers	Closed-loop supply chain	Examining the differing impacts of various recycling cost allocation methods on economic, environmental, and social benefits
Rao et al. [5]	Multi-brand EV manufacturers	Collaborative recycling perspective	Establishing a collaborative recycling alliance for EV manufacturers
Li et al. [6]	EV manufacturers, Retailers, Recycles	Characteristics of consumer heterogeneity	Optimal recycling strategies and profit levels for members of closed-loop supply chains under different recycling models
Feng et al. [7]	Two types of EV manufacturers with differentiated technologies	Market competition and Co-opetition Scenarios	Analysis of optimal recycling strategies for end-of-life batteries under competitive, patent-cooperative, and wholesale-cooperative models
Sun et al. [8]	EV manufacturers, Retailers	Carbon Trading Policy	The impact of battery life and advertising effectiveness on the selection of recycling channels and optimal supply chain decisions
Feng et al. [9]	PB suppliers, EV manufacturers, Third-party recyclers	Blockchain technology applications	The impact of blockchain traceability technology on battery production, sales, recycling decisions, and member profits
Zhang et al. [10]	PB manufacturers, EV manufacturers, Third-party recyclers	Carbon emissions trading policy	An analysis of optimal supply chain strategies and recycling model selection across four mixed recycling channels

**Table 2:** The Existing PFS distance measures.

Reference	PFS Distance Measures
Guong [11]	$d_{1}\left(ℑ,ℜ\right)=\frac{1}{n}\sum_{i=1}^{n}\left(\left|\xi_{ℑ}\left(x_{i}\right)-\xi_{ℜ}\left(x_{i}\right)\right|+\left|\psi_{ℑ}\left(x_{i}\right)-\psi_{ℜ}\left(x_{i}\right)\right|+\left|\vartheta_{ℑ}\left(x_{i}\right)-\vartheta_{ℜ}\left(x_{i}\right)\right|\right)$
Guong [11]	$d_{2}\left(ℑ,ℜ\right)=\sqrt{\frac{1}{n}\sum_{i=1}^{n}\left({\left(\xi_{ℑ}\left(x_{i}\right)-\xi_{ℜ}\left(x_{i}\right)\right)}^{2}+{\left(\psi_{ℑ}\left(x_{i}\right)-\psi_{ℜ}\left(x_{i}\right)\right)}^{2}+{\left(\vartheta_{ℑ}\left(x_{i}\right)-\vartheta_{ℜ}\left(x_{i}\right)\right)}^{2}\right)}$
Dinh and Thao [53]	$d_{3}\left(ℑ,ℜ\right)=\frac{1}{n}{\left\{\sum_{i=1}^{n}\left[{\left(\xi_{ℑ}\left(x_{i}\right)-\xi_{ℜ}\left(x_{i}\right)\right)}^{2}+{\left(\psi_{ℑ}\left(x_{i}\right)-\psi_{ℜ}\left(x_{i}\right)\right)}^{2}+{\left(\vartheta_{ℑ}\left(x_{i}\right)-\vartheta_{ℜ}\left(x_{i}\right)\right)}^{2}\right]\right\}}^{\frac{1}{2}}$
Dinh and Thao [53]	$d_{4}\left(ℑ,ℜ\right)=\frac{1}{n}\sum_{i=1}^{n}\max\left(\left|\xi_{ℑ}\left(x_{i}\right)-\xi_{ℜ}\left(x_{i}\right)\right|,\left|\psi_{ℑ}\left(x_{i}\right)-\psi_{ℜ}\left(x_{i}\right)\right|,\left|\vartheta_{ℑ}\left(x_{i}\right)-\vartheta_{ℜ}\left(x_{i}\right)\right|\right)$
Dinh and Thao [53]	$d_{5}\left(ℑ,ℜ\right)=\frac{1}{n}{\left[\sum_{i=1}^{n}\max\left({\left|\xi_{ℑ}\left(x_{i}\right)-\xi_{ℜ}\left(x_{i}\right)\right|}^{2},{\left|\psi_{ℑ}\left(x_{i}\right)-\psi_{ℜ}\left(x_{i}\right)\right|}^{2},{\left|\vartheta_{ℑ}\left(x_{i}\right)-\vartheta_{ℜ}\left(x_{i}\right)\right|}^{2}\right)\right]}^{\frac{1}{2}}$
Dinh and Thao [53]	$d_{6}\left(ℑ,ℜ\right)=\frac{1}{3n}\sum_{i=1}^{n}\left(\left|\xi_{ℑ}\left(x_{i}\right)-\xi_{ℜ}\left(x_{i}\right)\right|+\left|\psi_{ℑ}\left(x_{i}\right)-\psi_{ℜ}\left(x_{i}\right)\right|+\left|\vartheta_{ℑ}\left(x_{i}\right)-\vartheta_{ℜ}\left(x_{i}\right)\right|\right)$
Dutta [54]	$d_{7}\left(ℑ,ℜ\right)=\frac{1}{2}\sum_{i=1}^{n}\left(\left|\xi_{ℑ}\left(x_{i}\right)-\xi_{ℜ}\left(x_{i}\right)\right|+\left|\psi_{ℑ}\left(x_{i}\right)-\psi_{ℜ}\left(x_{i}\right)\right|+\left|\vartheta_{ℑ}\left(x_{i}\right)-\vartheta_{ℜ}\left(x_{i}\right)\right|+\left|\pi_{ℑ}\left(x_{i}\right)-\pi_{ℜ}\left(x_{i}\right)\right|\right)$
Dutta [54]	$d_{8}\left(ℑ,ℜ\right)=\frac{1}{2n}\sum_{i=1}^{n}\left(\left|\xi_{ℑ}\left(x_{i}\right)-\xi_{ℜ}\left(x_{i}\right)\right|+\left|\psi_{ℑ}\left(x_{i}\right)-\psi_{ℜ}\left(x_{i}\right)\right|+\left|\vartheta_{ℑ}\left(x_{i}\right)-\vartheta_{ℜ}\left(x_{i}\right)\right|+\left|\pi_{ℑ}\left(x_{i}\right)-\pi_{ℜ}\left(x_{i}\right)\right|\right)$
Dutta [54]	$d_{9}\left(ℑ,ℜ\right)=\frac{1}{2}\sum_{i=1}^{n}\left({\left|\xi_{ℑ}\left(x_{i}\right)-\xi_{ℜ}\left(x_{i}\right)\right|}^{2}+\left|\psi_{ℑ}\left(x_{i}\right)-\psi_{ℜ}\left(x_{i}\right)\right|+{\left|\vartheta_{ℑ}\left(x_{i}\right)-\vartheta_{ℜ}\left(x_{i}\right)\right|}^{2}+{\left|\pi_{ℑ}\left(x_{i}\right)-\pi_{ℜ}\left(x_{i}\right)\right|}^{2}\right)$
Dutta [54]	$d_{10}\left(ℑ,ℜ\right)=\frac{1}{2n}\sum_{i=1}^{n}\left({\left|\xi_{ℑ}\left(x_{i}\right)-\xi_{ℜ}\left(x_{i}\right)\right|}^{2}+{\left|\psi_{ℑ}\left(x_{i}\right)-\psi_{ℜ}\left(x_{i}\right)\right|}^{2}+{\left|\vartheta_{ℑ}\left(x_{i}\right)-\vartheta_{ℜ}\left(x_{i}\right)\right|}^{2}+{\left|\pi_{ℑ}\left(x_{i}\right)-\pi_{ℜ}\left(x_{i}\right)\right|}^{2}\right)$

**Table 3:** Robustness metrics of PFS distance measure under noise disturbance.

PFS Distance Measure	Mean	Standard Deviation	Coefficient of Variation
$d_{1}\left(ℑ,ℜ\right)$	0.1682	0.0239	0.1419
$d_{2}\left(ℑ,ℜ\right)$	0.1945	0.0246	0.1264
$d_{3}\left(ℑ,ℜ\right)$	0.1945	0.0246	0.1264
$d_{4}\left(ℑ,ℜ\right)$	0.2151	0.0278	0.1294
$d_{5}\left(ℑ,ℜ\right)$	0.0384	0.0098	0.2538
$d_{6}\left(ℑ,ℜ\right)$	0.5047	0.0716	0.1419
$d_{7}\left(ℑ,ℜ\right)$	0.0841	0.0119	0.1447
$d_{8}\left(ℑ,ℜ\right)$	0.2012	0.0291	0.1447
$d_{9}\left(ℑ,ℜ\right)$	0.2270	0.0348	0.1532
$d_{10}\left(ℑ,ℜ\right)$	0.1135	0.0174	0.1532
$d\left(ℑ,ℜ\right)$	0.3302	0.0391	0.1185

**Table 4:** PF decision matrix Z.

Alternative	Evaluation Attribute				
	*C*_1_	*C*_2_	*C*_3_	*C*_4_	*C*_5_
*A*_1_	(0.63, 0.21, 0.14)	(0.55, 0.29, 0.12)	(0.78, 0.12, 0.09)	(0.64, 0.18, 0.12)	(0.57, 0.17, 0.20)
*A*_2_	(0.77, 0.12, 0.10)	(0.69, 0.18, 0.09)	(0.70, 0.18, 0.11)	(0.56, 0.29, 0.12)	(0.63, 0.15, 0.11)
*A*_3_	(0.63, 0.18, 0.12)	(0.76, 0.12, 0.09)	(0.79, 0.09, 0.10)	(0.81, 0.09, 0.09)	(0.74, 0.12, 0.09)
*A*_4_	(0.69, 0.18, 0.10)	(0.65, 0.22, 0.11)	(0.57, 0.28, 0.12)	(0.65, 0.21, 0.11)	(0.48, 0.31, 0.18)

**Table 5:** PISs and NISs for each evaluation attribute.

Evaluation Attribute	$v_{j}^{+}$	$v_{j}^{-}$
$C_{1}$	(0.77, 0.12, 0.10)	(0.63, 0.21, 0.14)
$C_{2}$	(0.76, 0.12, 0.09)	(0.55, 0.29, 0.12)
$C_{3}$	(0.79, 0.09, 0.09)	(0.57, 0.28, 0.12)
$C_{4}$	(0.81, 0.09, 0.09)	(0.56, 0.29, 0.12)
$C_{5}$	(0.74, 0.12, 0.09)	(0.48, 0.31, 0.20)

**Table 6:** Distances between each alternative and the PIS.

Alternative	PIS of Each Attribute				
	$v_{1}^{+}$	$v_{2}^{+}$	$v_{3}^{+}$	$v_{4}^{+}$	$v_{5}^{+}$
$A_{1}$	0.0457	0.0708	0.0068	0.0501	0.0581
$A_{2}$	0.0000	0.0224	0.0340	0.0816	0.02897
$A_{3}$	0.03817	0.0000	0.0017	0.0000	0.0000
$A_{4}$	0.02380	0.0393	0.0756	0.0510	0.0937

**Table 7:** Distances between each alternative and the NIS.

Alternative	NIS of Each Attribute				
	$v_{1}^{-}$	$v_{2}^{-}$	$v_{3}^{-}$	$v_{4}^{-}$	$v_{5}^{-}$
$A_{1}$	0.0000	0.0000	0.0680	0.0331	0.0397
$A_{2}$	0.0457	0.0486	0.0408	0.0000	0.0709
$A_{3}$	0.0087	0.0708	0.0735	0.0816	0.0962
$A_{4}$	0.0222	0.0319	0.0000	0.0309	0.0034

**Table 8:** Weighted total distances of the four alternatives.

	Weighted Total Distance	$D_{1}^{+}$	$D_{1}^{-}$
Alternative			
$A_{1}$		0.0477	0.0248
$A_{2}$		0.0324	0.0402
$A_{3}$		0.0087	0.0636
$A_{4}$		0.0529	0.0193

**Table 9:** Ranking results of different picture fuzzy distance.

Distance Measure	Relative Proximity				Ranking Result
	$RC_{1}$	$RC_{2}$	$RC_{3}$	$RC_{4}$	
$d_{1}$	0.3431	0.5524	0.8808	0.2674	$A_{3}>A_{2}>A_{1}>A_{4}$
$d_{2}$	0.3552	0.5363	0.8725	0.2702	$A_{3}>A_{2}>A_{1}>A_{4}$
$d_{3}$	0.3552	0.5363	0.8725	0.2702	$A_{3}>A_{2}>A_{1}>A_{4}$
$d_{4}$	0.3528	0.5305	0.8520	0.2755	$A_{3}>A_{2}>A_{1}>A_{4}$
$d_{5}$	0.3528	0.5305	0.8520	0.2755	$A_{3}>A_{2}>A_{1}>A_{4}$
$d_{6}$	0.3431	0.5524	0.8808	0.2674	$A_{3}>A_{2}>A_{1}>A_{4}$
$d_{7}$	0.3258	0.5497	0.8587	0.2758	$A_{3}>A_{2}>A_{1}>A_{4}$
$d_{8}$	0.3528	0.5497	0.8587	0.2758	$A_{3}>A_{2}>A_{1}>A_{4}$
$d_{9}$	0.3472	0.5083	0.9238	0.1459	$A_{3}>A_{2}>A_{1}>A_{4}$
$d_{10}$	0.3472	0.5083	0.9238	0.1459	$A_{3}>A_{2}>A_{1}>A_{4}$
d	0.3423	0.5536	0.8796	0.2673	$A_{3}>A_{2}>A_{1}>A_{4}$

## Data Availability

All used data included in the manuscript.

## Abbreviations

The following abbreviations are used in this manuscript:

EV	Electric vehicle
PB	Power battery
DM	Decision-making
MADM	Multi-attribute decision-making
PFS	Picture fuzzy set
IFS	Intuitionistic fuzzy set
PF	Picture fuzzy
TOPSIS	Technique for order preference by similarity to ideal solution
PIS	Positive ideal solution
NIS	Negative ideal solution
